# Is There an Association between Executive Function and Receptive Vocabulary in Bilingual Children? A Longitudinal Examination

**DOI:** 10.3390/children8010044

**Published:** 2021-01-13

**Authors:** Vanessa Diaz, Maria Borjas, M. Jeffrey Farrar

**Affiliations:** 1 Department of Psychology, Virginia Tech, Blacksburg, VA 24061, USA; 2 Department of Psychology, The University of Houston, 3695 Cullen Boulevad, Houston, TX 77204, USA; mborjas021@gmail.com; 3 Department of Psychology, University of Florida, Gainesville, FL 32611, USA; farrar@ufl.edu

**Keywords:** bilingualism, executive functioning, receptive vocabulary, language development, longitudinal

## Abstract

Dual language management has been proposed as the reason for bilingual children’s sometimes enhanced executive functioning (EF). We sought to identify the directionality of the relation between language proficiency and EF, using measures of receptive vocabulary, inhibitory control, and cognitive flexibility. Data were collected twice, a year apart, on 35- to 66.8-month-old bilingual (*n* = 41, *M* = 49.19 months) and monolingual preschool children (*n* = 37, *M* = 47.82 months). The longitudinal results revealed that while the monolingual children’s vocabulary at Time 1 predicted EF at Time 2, EF at Time 1 did not predict vocabulary at Time 2. In contrast, for bilingual children the relation was not present at all. The results were similar after the one-time analyses. The absence of relations between EF and language in bilinguals, while present in monolinguals, challenges the current conceptualization of the EF advantage in bilinguals, and emphasizes the need for more research on the development of bilingual children.

## 1. Introduction

The specific nature of the relation between language development and executive functioning (EF) has been much examined and greatly debated (e.g., [1,2,3]). EF refers to higher-order cognitive processes such as attention management, planning, monitoring, and inhibition of habitual responses [1]. Previous longitudinal studies on this topic on monolingual children have found varying associations in terms of directionality, suggesting a number of possible underlying mechanisms. These suggested mechanisms include simultaneous and interactive development [3]; primacy of EF relative to language development [4]; the influence of a potential third factor, such as processing speed [5,6]; and that language proficiency may act as a mediator between EF and other factors, such as phonological awareness. Other studies have found that language proficiency mediates EF group differences but not the reverse [7], or in the case of Slot and Suchodoletz (2018) [3], that even when the relationship is bidirectional, earlier language proficiency is a stronger predictor of later EF rather than the reverse. We will review these findings in greater detail, as well as the available findings on bilingual populations.

For bilingual children, determining the directionality of the relation between receptive vocabulary skills, a measure of language proficiency, and EF is particularly interesting in light of the accumulating evidence showing differential development between bilingual and monolingual children on a number of related socio-cognitive and psycholinguistic capacities. For example, we see performance differences between bilingual and monolingual children in theory of mind development (i.e., [8]), metalinguistic awareness [9], as well as on EF [10] and language development themselves [11]. In this paper, we are interested in the following question: Does potentially differential development in EF and receptive vocabulary compared to monolingual children result in a comparatively different relation between them in bilingual children?

Specifically, a number of studies have found that bilingual children have an advantage when it comes to EF compared to monolingual children [12,13,14,15,16], although this has been recently challenged (see [17,18]). This potential EF advantage in bilinguals has been proposed as resulting from the nature of bilingual language use (i.e., [10]), which requires efficient language monitoring, selection, inhibition of the non-selected language, and cognitive flexibility to switch as appropriate, thus resulting in a strengthened EF system [19]. A bilingualism effect on EF has been reported in children of different ages and cultural backgrounds, such as 8-year-old Canadian and Indian bilingual compared to monolingual Canadian children [10], Spanish–English bilinguals compared to English monolinguals, and English speakers enrolled in a second-language immersion kindergarten [1], as well as in toddlers [15] and infants [20]. On the other hand, bilingual children have also been shown to underperform in language proficiency, such as measures of receptive vocabulary, a difference that has been shown to be stable over time [11], though these assessments are usually limited to receptive vocabulary in their dominant language. Given the characterization in previous research that the bilingual EF enhancement on cognitive flexibility and inhibitory control comes from using these capacities to develop proficiency in two languages, here we ask instead if the reverse could also be the case, could a strengthened EF lead to improved language outcomes?

Previous longitudinal studies on the relation between EF and language proficiency in monolingual children have produced several findings. In a recent longitudinal study with measurements taken over a one-year period, language skills, assessed through receptive vocabulary and the comprehension and imitation of grammatical structures, were a significant predictor of later EF performance for monolingual three- to four-year-old children [3]. The authors used the Pencil Tapping task, Dimensional Change Card Sort (DCCS) task, Forward Digit Span, and Copy Hand Movement task as their EF measures to assess inhibitory control, attention shifting, and working memory. Although this relation was bidirectional, language skills were a stronger predictor of later EF performance than EF was of later language skills [3]. Based on this finding, the authors proposed that the nature of a child’s language development may suggest that language skills and EF skills develop simultaneously and interactively. In contrast, Weiland and colleagues (2014) [4] reported a different pattern of relations in a sample of monolingual children. In their longitudinal study, the authors found that EF skills at Time 1 predicted receptive vocabulary at Time 2 and, unlike Slot and Suchodoletz [3], they did not find this relation to be bidirectional. The authors argued that the direction of this relationship is intuitive due to the fact that preschool curricula tend to target receptive vocabulary, with not much emphasis on EF, thus allowing EF to develop on its own [4]. It is worth noting that Weiland et al. (2013) used the Pencil Tap task, Forward Digit Span task, and Backward Digit Span task to measure EF, and the Peabody Picture Vocabulary Test to measure receptive vocabulary, similarly to Slot and Suchodoletz [3]. Kaushanskaya and colleagues [21] reported a similar finding with an older sample of eight- to ten-year-old monolingual children. They assessed EF using the Flanker task, Go/No-Go task, n-back task, Corsi Block task, and the DCCS to measure inhibition, working memory, and task shifting; and comprehensive language assessments, including receptive vocabulary and the Clinical Evaluation of Language Fundamentals (CELF). The authors reported a predictive relationship between working memory and language proficiency, but there was an overall weak relation between language scores and other aspects of EF, such as inhibition and shifting [21].

For a different pattern of results, another longitudinal study on monolingual children in which data were collected at ages 4, 5, 6, and 7, a stable relation between language and EF was found at each time point, but interestingly, no directional association over time was found between the two factors [5]. The tasks used to assess EF included the Head Toes Knees and Shoulders task, Block Recall task, and the Go/No-Go task to measure inhibition, selective attention, and visuo-spatial memory. Language was assessed using the Receptive One Word Picture Vocabulary Test (ROWPVT), Experimental Sentence Imitation Task, and two subsets (sentence structure and expressive vocabulary) of the CELF. The authors argue that these results may be due to a third factor, such as processing speed, influencing both. In contrast, a study with a sample composed of deaf and hearing children found that language mediated non-verbal EF differences between the groups, but that non-verbal EF performance did not mediate language group differences [7]. The authors used a long array of executive function measures, including the Odd One Out Span task, Backward Spatial Span task, Design Fluency task, Children’s Color Trails Tests 1 and 2, Tower of London task, and (computerized) Simon task to assess visuospatial working memory and cognitive fluency, shifting, planning, and inhibitory control. The Expressive One-Word Picture Vocabulary Test (EOWPVT) was used to assess language skills. These results suggest that in a sample with non-normative language development, language proficiency became more influential. For a different kind of language measure, Wilbourn and colleagues [6] looked at the relation between phonological awareness, receptive vocabulary, and EF (assessed using the Lexical Stroop task and the DCCS task to assess cognitive flexibility and attention) in a sample of five- to eight-year-old English monolinguals. They proposed that vocabulary proficiency may act as a mediator in the relation between performance on an EF task and phonological awareness, suggesting a much more intricate relation between language development and EF.

Overall, studies on monolingual children have found support for all three longitudinal relationships: EF predicting language proficiency, language proficiency predicting EF, and bidirectional relations. Taken together, we propose that this likely points to the relationship between EF and receptive vocabulary skills, an aspect of language proficiency, at least in monolingual children, as being simultaneous, interactive, and also bidirectional.

As reviewed, the majority of our current understanding of the longitudinal relation between EF and language development comes from monolingual samples. When it comes to bilingual children, a number of researchers have examined this question with the purpose of finding out whether and when there is a bilingual advantage on EF [12,13,14,15]. For example, in the only longitudinal study of this kind, the authors found an inhibitory control EF advantage for bilinguals when testing two- to three-year-old monolinguals and bilinguals at six months apart [22]. Specifically, knowledge of translation equivalents assessed through a parental report vocabulary checklist was associated with better performance on the EF tasks assessing cognitive flexibility, working memory, inhibitory control, and response suppression (Reverse Categorization task, Shape Stroop task, Gift Delay task, and the Multilocation task), suggesting a connection between proficiency on both languages and EF [22]. Importantly, however, the directionality of this relationship was not examined.

When it comes to single time point studies examining the relation between language development and EF in bilinguals, Carlson and Meltzoff [1] found enhanced EF abilities for bilinguals related to specific language exposure. The authors found an EF advantage for five- to seven-year-old bilingual children compared to both monolinguals and children in an immersion program on the battery of conflict EF tasks compared to the battery of EF delay tasks. In turn, Iluz-Cohen and Armon-Lotem [23] found that four- to seven-year-old bilingual children with high language proficiency performed better at tasks assessing inhibition and shifting than participants with low language proficiency, as measured by the Goralnik Diagnostic Test for Hebrew and the CELF. So, while some studies have focused on the nature of the relation between language proficiency and EF for this group, there are no longitudinal investigations that draw their attention to the directionality of these relations in bilingual children.

In the current investigation we examine the directionality of this relationship across time in bilinguals and monolinguals to answer the following questions: Does more efficient EF at one point predict improved language proficiency a year later as measured by receptive vocabulary? Does higher language proficiency, as measured by receptive vocabulary, instead predict a more efficient EF later on? Is the relation bidirectional? To our knowledge, our study is the first to examine the longitudinal relation between EF and receptive vocabulary skills in bilingual preschool children. Given the variations that exist between language and EF for bilinguals and monolinguals, a longitudinal analysis may enhance our understanding of the relation between EF and language for the two groups.

As described, the motivation for examining this question in bilingual children regards the cognitive flexibility and inhibitory control required to manage dual linguistic representations by inhibiting one language and actively selecting another. What would the pattern of a single time point and longitudinal associations be in this group given this particular need, as well as the pattern of relations described above for monolingual children? For bilingual children we see two possibilities. On the one hand, effective learning of two languages as reflected in vocabulary scores at one time may predict later enhanced EF through a generalization of the control mechanism, or language learning as a tool for attention control. On the other hand, stronger EF at one point may in turn facilitate subsequent bilingual language development (demonstrated in vocabulary scores) by facilitating the management of dual linguistic representations, thus enhancing cognitive flexibility and inhibition, as well as receptive vocabulary skills. Both of these possibilities carry theoretical significance, firstly for our understanding of the relation between language development and EF, and secondly for our understanding of bilingual development.

### Current Study

In the present study, we aim to identify the directionality of the relation between language proficiency, as measured through receptive vocabulary, and EF using measures of inhibitory control and cognitive flexibility, in samples of monolingual and bilingual children using two time points a year apart. We are interested in analyzing whether receptive vocabulary skills predict EF in monolingual and bilingual children and/or whether EF, in turn, predicts receptive vocabulary skills. The absence in the literature on this relationship, focusing on bilingual children specifically, leaves an important gap in our understanding of bilingual children’s development. We are particularly interested in possible differences in these relations for bilingual and monolingual children, which may suggest differences in the mechanisms underlying development in these two groups.

Our specific research questions are as follows: (1) Does a longitudinal relation exist between language proficiency, as measured by receptive vocabulary, and EF, similar to the literature on monolingual children? If so, does this measure of language proficiency at Time 1 predict EF at Time 2, and/or does EF at Time 1 predict receptive vocabulary assessed at a Time 2? (2) Are there identifiable differences in the directionality of the relation between receptive vocabulary skills and EF for monolingual and bilingual children? We use receptive vocabulary as our language proficiency measure because it captures the understanding of linguistic labels that young language learners may not yet feel comfortable producing, which is particularly relevant for dual language learners. Moreover, we theorize that bilingual learners would engage EF resources to manage dual linguistic representations as denoted by their total vocabulary knowledge, which includes understanding of labels in both languages. We selected EF tasks as well-established conflict measures tapping into inhibitory control and cognitive flexibility.

## 2. Materials and Methods

### 2.1. Participants

Our sample was composed of 41 Spanish–English bilingual children and 37 English monolinguals. Their ages ranged from 35 to 66.8 months at Time 1 (Bilinguals *M* = 49.19 months, *SD* = 7.32; Monolinguals *M* = 47.82 months, *SD* = 6.94). Because of this range, we controlled for age on all of our analyses. Participants were recruited from predominantly bilingual and monolingual preschools in the Southeastern United States, and this protocol was approved by the University of Florida’s Institutional Review Board (UFIRB #2011-U-451). Bilingualism was defined by regular exposure to both English and Spanish, and parental reports of fluency in both languages. This was assessed through parent language questionnaires where parents were asked “1. Which language/languages does your child speak (may list more than one)?”; “2. Which one is your child’s preferred language?”; “3. Which language/languages are regularly spoken in your home?”; and “4. Which language/languages is the child exposed to in Preschool?”—among other language-exposure questions. Only children whose parents listed two languages for Question 1 and for either Questions 3 or 4 were considered bilinguals. All such parents listed Spanish and English for Question 1. Of this sample of Spanish–English bilinguals, a large majority (77.5%) were exposed to both languages since birth (see Table 1). The non-crib bilinguals had all been exposed to their second language for one year or more.

No significant differences were found between the bilingual and monolingual groups on gender and SES (parental marital status, and the education, employment, and occupational status of both parents), nor for the primary caregiver’s level of education (all *p*-values > 0.05; presented in Table 3). A year later, the children were assessed again. The final sample was composed of 25 bilinguals and 27 monolingual children (Bilinguals *M* = 56.97 months, *SD* = 5.25; Monolinguals *M* = 57.22 months, *SD* = 6.68). Because testing was conducted over the summer months, attrition was due to the children moving away or changing schools, and was not significantly different for the bilingual and monolingual groups, thus not systematic for our main comparisons. In addition, we ran comparisons on the demographic variables and our main variables of interest at Time 1 between those children who returned for Time 2 and those who did not. We found no significant differences between the return and no-return sample on gender and parental education. We did find differences for age (*t*(76) = 2.84, *p* < 0.01) and SES (*t*(69) = −2.47, *p* < 0.05). The no-return sample was older (return *M* = 46.82, *SD* = 5.80 vs. no-return *M* = 51.98, *SD* = 8.32) and had lower SES. These differences lead us to speculate that the older and lower-income children were more likely to start attending Voluntary Prekindergarten (VPK), a free educational program for 4-year-olds in the region. In terms of our variables of interest (EF and vocabulary), once controlling for differences in age, comparisons between the return and no-return sample revealed no differences. These results give us confidence to report on the entire Time 1 sample, as well as on the return sub-sample.

### 2.2. Vocabulary Measure

Receptive One Word Picture Vocabulary Test [24]: The ROWPVT is normed for examinees 2–80 years of age and has a median reliability of 0.97 across all ages. Children are shown test plates with four pictures each and asked to point to the picture that best represents a word spoken by the experimenter. Testing begins with words normed on the child’s chronological age and increases in difficulty until eight consecutive errors are made. The test is scored by deducting errors from the number of the final item administered.

The English monolingual version was used for the monolingual children at the beginning of the testing session, followed by the EF measures. For bilingual children a Spanish–English bilingual version of the test was used to assess proficiency in both languages using the same test plates. The test was first administered in the child’s non-preferred language, as reported in the parent language questionnaire (Table 1), because the test stimuli were the same for both languages. The test was then re-administered in their preferred language at the end of the testing session, after the EF measures. For children whose parents listed that the child had no preference, the child was asked for their preference to conduct the session at the time of testing and followed the procedure above. The testing then proceeded with the EF measures in the child’s preferred language. At the end of the session, the vocabulary test was re-administered in the children’s preferred language by the same research assistant who had native-like proficiency in both languages. Results for the bilingual children were tallied for both a conceptual score and a total score, as described below.

Conceptual Score: A conceptual score was computed by adding the total number of concepts the children pointed to correctly in either one or both languages. This effectively assessed the number of “concepts” the children recognized, regardless of the language used by the investigator. This is thought to more adequately represent their language competence versus only analyzing their dominant language [25] compared to monolinguals.

Total Score: We computed a total language score by adding the bilingual children’s separate scores in both Spanish and English. While it may not always be appropriate to use this score in comparisons with monolinguals, because it counts translation equivalents as two individual items, we were interested in whether competency in both languages may be a good measure of EF’s involvement in the bilingual’s effective acquisition of two lexicons. These values are presented in Table 2.

### 2.3. Executive Functioning Measures

Executive functioning was assessed using the following several measures focusing on inhibitory control and cognitive flexibility. A composite score was created by obtaining the z-score of each task due to differing number of items in each and adding these z-scores together. Individual EF task group scores and correlations with vocabulary are reported in Table 2 and Table 3.

Bear/Dragon Task [26]: For this inhibitory control and cognitive flexibility Simon-Says like measure, children were asked to follow the commands of a bear puppet and ignore the commands of a dragon puppet. The children received points for ignoring the dragon’s command, and for following the bear’s commands. There were 10 trials split evenly and alternating between the bear commands and dragon commands. This task had a total of 10 possible points.

Happy/Sad Task [27]: For this inhibitory control measure, children were asked to say “happy” when shown a picture of a “sad” face, and “sad” when shown a picture of a “happy” face. The child was given a point for every correct response. There were 16 trials split evenly between “happy” and “sad” faces in random order. This task has a total of 16 possible points.

Dimensional Change Card Sort Task (DCCS) [28]: For this cognitive flexibility measure, the child was asked to sort pictures of red or blue boats and rabbits by color and then by shape. The child was given a point for every picture that was correctly sorted. This task had a total of 6 possible points. Only the switched trials were scored.

### 2.4. General Procedure

The data presented here were obtained as part of a larger study assessing the cognitive development in bilingual children. At Time 1, consent was obtained from the parents of the participants through signed letters returned to the children’s school with the demographic and language exposure questionnaires. Parents were compensated for filling out the questionnaires. The testing sessions lasted about 45 min and were conducted in quiet offices or extra rooms at the children’s preschools. Testing was conducted in English for English monolinguals and in the language selected by the parent as the preferred language for bilingual children. This preference was corroborated by both the children and their teachers. Time 2 data were collected about a year later, using the same procedure.

## 3. Results

Analyses were first conducted to detect possible demographic differences between the monolingual and bilingual children. No significant differences were found between bilinguals and monolinguals on age, gender, parental level of education, and SES for the total sample at Time 1, nor for the return sample (all *p*-values > 0.05). In addition, we performed regression analyses for the receptive vocabulary scores based on SES for both groups combined, and separately for monolinguals and bilinguals using both the total and conceptual scores controlling for age (Time 1 data). The analyses revealed a non-significant effect of SES for both groups together, using the conceptual score for bilinguals (β = 0.20 *t*(68) = 1.85, *p* = 0.068) and for each group separately (monolinguals β = 0.07 *t*(31) = 0.45, *p* = 0.65; bilingual conceptual β = 0.20 *t*(34) = 1.37, *p* = 0.17; bilingual total β = 0.22 *t*(34) = 1.50, *p* = 0.14).

We then examined the receptive vocabulary and EF performance differences between the groups, and the relation between receptive vocabulary and EF performance at Time 1, Time 2, and between Time 1 and Time 2, using partial correlations controlling for age and for autoregressive effects.

### 3.1. Time 1 and Time 2

Independent sample t-tests between bilinguals and monolinguals on EF at both time points revealed only differences on the DCCS task at Time 2, with monolinguals outperforming bilinguals at *t*(50) = 3.53, *p* = 0.001, η2 = 0.20 (see Table 3). For vocabulary, in contrast, we found that monolinguals outperformed bilinguals at both Time 1 (*t*(76) = 5.49, *p* = 0.000, η2 = 0.28) and Time 2 (*t*(50) = 4.36, *p* = 0.000, η2 = 0.27) using the conceptual score for bilinguals. In turn, bilinguals outperformed monolinguals when using the total score, which added their Spanish and English vocabulary scores (Time 1 (*t*(76) = −2.42, *p* = 0.018 and Time 2 (*t*(50) = −3.13, *p* = 0.004). The total scores are a holistic reflection of language competence in bilinguals and demonstrated no language deficiencies when knowledge of their both languages is considered. These comparisons remained the same when only considering the Time 2 return sample at Time 1.

The main focus of this investigation was the relation between language proficiency and EF for each group. Starting with each time point separately, at Time 1, controlling for age, receptive vocabulary was correlated with the EF composite (*r*(34) = 0.41, *p* = 0.014), but only for the monolingual children (see Table 2). For bilinguals, the correlation between receptive vocabulary and EF was not significant, using both the conceptual score (*r*(37) = 0.15, *p* = 0.158) and the total score. For the return sample only, the results were similar with only a significant correlation between receptive vocabulary and EF for the monolingual sample (*r*(24) = 0.47, *p* = 0.018). Similar to Time 1, for Time 2, also controlling for age, there was a significant relation between receptive vocabulary and EF for the monolingual group (*r*(23) = 0.50, *p* < 0.05), but not for the bilingual group using either the conceptual or total language score for bilinguals. All Time 2 and longitudinal analyses include the return sample only.

### 3.2. Longitudinal Relations

We were particularly interested in analyzing the longitudinal directionality of the relation between receptive vocabulary and EF by using cross-lagged correlation analyses. Specifically, we were interested in which factor (vocabulary, EF) at Time 1 predicted performance on the other factor at Time 2, and in identifying any differences between the groups in these patterns. As in the previous analyses, we controlled for age at both time points. In addition, we also controlled for the effect of the target Time 2 variable at Time 1 to account for autoregressive effects (see Table 4).

**Figure 1:**
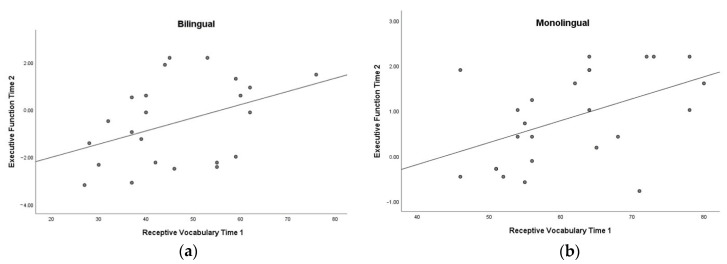
Relation between receptive vocabulary at Time 1 and EF at Time 2 for bilinguals (**a**) and monolinguals (**b**).

For monolinguals, similar to their single time point results, we found that receptive vocabulary at Time 1 was related to EF at Time 2, (*r*(21) = 0.58, *p* = 0.004). However, EF at Time 1 was not related to receptive vocabulary at Time 2 (*r*(21) = 0.16, *p* = 0.602). This suggests that, while for monolinguals language proficiency is significantly predictive of later EF, this relation was not bidirectional. For bilinguals, in contrast, using the receptive conceptual vocabulary score no longitudinal relation was significant; that is, in contrast to our monolingual sample, Time 1 receptive vocabulary did not predict EF at Time 2 for bilinguals (*r*(18) = −0.08, *p* = 0.728), nor did EF at Time 1 predict receptive vocabulary at Time 2 (*r*(17) = 0.09, *p* = 0.708). Using the total vocabulary scores for the bilingual sample did not change the findings (see Table 4). These differences can be observed in Figure 1, where a linear trend between receptive vocabulary at Time 1 and EF can be observed for monolinguals but not for bilinguals. We corroborated these results by running repeated measures ANCOVAs for each group and the EF Time 2 composite as the outcome variable. Receptive vocabulary at Time 1 significantly predicted EF at Time 2, controlling for EF at Time 1 and age at both time points for the monolingual group only (*F* (1, 25) = 8.56, *p* = 0.004, η2p = 0.95).

## 4. Discussion

For this investigation, we were interested in the relation between two centrally situated cognitive development skills: EF and language proficiency. We analyzed receptive vocabulary, a measure of language proficiency, and EF across two time points a year apart for English monolingual and Spanish–English bilingual children. Previous studies have identified an EF advantage for bilinguals, often hypothesized to emerge from their effective language management [1,13,14,15,22]. Following this line of reasoning, we hypothesized that a relation should exist between receptive vocabulary and EF for bilingual children. One such relation may involve higher EF, leading to more extensive vocabularies through the effective management of dual linguistic representations as reflected in our total language score, where translation equivalents were counted individually. We also hypothesized that more receptive vocabulary skills could in turn lead to higher EF through a strengthening of control mechanisms that bilinguals may employ during dual language processing and acquisition. A third possibility involved both of these relations through bidirectional longitudinal relations. In the monolingual literature there is support for all three directions of effect between EF and vocabulary acquisition.

For our bilingual sample, we did not find any of the above scenarios. Results of the single time point and longitudinal cross-lagged analysis revealed a relation between receptive vocabulary and EF measures, but only for monolingual children. At both Time 1 and Time 2, receptive vocabulary was significantly correlated with EF only for monolinguals. Importantly, receptive vocabulary at Time 1 predicted EF at Time 2. When controlling for autoregressive effects, the relation was not found to be bidirectional for the monolingual group. These findings support those of previous studies that have identified a significant relation between receptive vocabulary and EF in monolingual children [3,4,5], especially those finding that receptive vocabulary predicts EF in monolingual children rather than the reverse (i.e., [3,7]). In our sample, monolingual children’s language proficiency as measured by receptive vocabulary was predictive of EF performance a year later. A number of possibilities may account for this. In our case, as it is for most EF tasks, there is an important verbal component to the tasks, such as understanding the instructions and the prompts. Another possibility regards the connection between the internalization of private speech, language skills, self-regulation, and cognitive abilities [29,30]. It is possible, for example, that in our sample of monolingual children language skills at Time 1 were instrumental in the development of self-regulation and EF through Time 2. Regarding the non-significant relation between Time 1 EF and later language skills, we posit that perhaps as the instructional environment becomes more rigorous, EF skills become more instrumental for other types of knowledge acquisition than during the preschool years.

In contrast to the consistent findings on monolingual children, no significant relations were found for the bilingual group between receptive vocabulary and EF. This stark difference raises questions about both the established relation between linguistic processes and EF found in monolinguals in previous research in terms of its applicability for bilingual populations, as well as how to characterize the advantage that bilinguals have been reported to have when performing EF tasks (even though that was not the case in our sample). As described, this bilingual advantage is often credited to their effective language management. Given the lack of relation between EF and receptive vocabulary, even for the two languages combined for bilinguals in the current study, there remains a need to better characterize this phenomenon; that is, how effective bilingual children were at acquiring and recognizing labels for normed vocabulary words of the same objects in their two languages combined was not related to their EF skills, as it was for monolingual children in their one language. These results suggest the need for a closer analysis of the relation between language proficiency and EF, and the role these two variables play in children’s cognitive development.

In line with our results, a recent study by Nicoladis et al. [31] also failed to find a relation between EF cognitive flexibility, using the DCCS, and measures of either language dominance (parental reports of dominance, relative scores on vocabulary tests, and knowledge of translation equivalents) or language use (living in a bilingual or monolingual community and a language separation task) in French–English bilingual children in Montreal three to six years of age. In addition to the present study, this reiterates the need for future research on the development of bilingual children. We propose that, given the lack of relations detected in this sample, other variables related to language development and EF should be analyzed as part of alternative ways of conceptualizing the development of EF and language proficiency in bilingual children. For example, theory of mind (e.g., [8]) and metalinguistic awareness, such as syntactic awareness [9], have been shown to develop differently in bilingual children and may be related to how bilingual children are making sense of the particularly complex linguistic contexts they experience, instead of EF. Other cognitive skills that may be particularly relevant to bilinguals’ receptive vocabulary development (e.g., working memory, phonological awareness) may reveal different learning routes for bilingual vocabulary skills compared to those of monolinguals, and should be addressed by future studies. It follows, for example, that since the mapping between concepts and lexical items is one-to-one in monolinguals and one-to-two in bilinguals, different cognitive skills could be involved. A related issue is that some EF skills, such as inhibition, may be more related to productive measures and not receptive ones, due to the inhibition needed to select the language and produce a word. It is also important to expand this topic of research across different languages and regions. While our monolingual data was collected in Central Florida, the bilingual data was collected from Spanish–English bilinguals in South Florida. South Florida has its own contextual particularities, such as a high number of bilingual speakers (see Table 1), versus other bilingual contexts where the languages may be more compartmentalized. It is possible, for example, that in this context the children do not have to inhibit their language as often, thus accounting for the lack of bilingual advantage in EF; but also that their language learning environment is particularly challenging. This kind of sociolinguistic context may lead to a diminished interaction between EF and linguistic processes compared to other kinds of bilingual contexts where the languages are more compartmentalized than in this largely bilingual community. For example, if one language is spoken at home and a different language is spoken at school, children would need to recruit more EF resources in order to inhibit the “wrong” language, and switch to the appropriate one. In this bilingual sample, however, only 25% of parents reported that only English was spoken at their children’s school (see Table 1). This does not explain, however, why there would be a lesser relation compared to monolingual children. Other limitations of the current study include its relatively small sample size compounded by attrition, as addressed in our “Participants” section. In addition, there may be significant variation within our bilingual sample’s language exposure and language use, thus making correlations harder to detect. This may be particularly relevant coming from the parental language questionnaires, which may not be entirely accurate at capturing the important distinction between language use versus language exposure in bilinguals [32].

## 5. Conclusions

The current study further elucidates the relation between language proficiency, as measured by receptive vocabulary, and EF in monolingual children by demonstrating that future EF performance is predicted by previous language proficiency. This study also demonstrates that relations between EF and language in monolingual children, such as the one described, may not necessarily apply to bilingual children. This latter point brings awareness to the importance of continued research on the development of bilingual children. Our results were not congruent with the current conceptualization of the EF advantage in bilingual children found in other samples. In turn, we suggest that future research should examine alternative factors that may be supporting bilingual children’s linguistic development, perhaps using a larger sample than the one we were able to retain for the longitudinal analyses. What are bilingual children using to make sense of linguistic input and contexts where two languages are used simultaneous by either the same or different individuals, in the same or different contexts, and with more than likely different levels of proficiency?

## Figures and Tables

**Table 1:** Bilingual children’s language exposure parental questionnaire.

	% English Only	% Spanish Only	% Both
Language exposed at birth	6	15	77.5
Child’s preferred language	46.2	48.7	5.1
Spoken at home	12.5	55	27.5
Spoken at preschool	25.6	7.7	61.5
Language mother speaks to child	15.4	59	25.6
Language father speaks to child	10.3	53.8	30.8
Language other adult in the home speaks to child	-	66.7	33.3

**Table 2:** Partial correlations controlling for age for bilinguals and monolinguals between receptive vocabulary and executive functioning (total receptive vocabulary score between parentheses) at Time 1 and Time 2.

Receptive Vocabulary	Time 1		Time 2	
	Monolinguals	Bilinguals	Monolinguals	Bilinguals
Executive Function Composite	0.41 *	0.13 (0.15)	0.50 *	38 (0.37 ^)
Happy/Sad task	−0.04	−0.06 (−0.02)	0.52 **	0.27 (0.32)
Bear/Dragon task	0.53 *	0.19 (0.24)	0.44 *	0.08 (0.02)
Card Sort task	0.25	0.17 (0.14)	0.21	0.33 (0.25)

Note: ^ *p* < 0.10; * *p* < 0.05, ** *p* < 0.01.

**Table 3:** Bilingual and monolingual children’s mean performance (standard deviation in parentheses) and group differences at Time 1 and Time 2.

	Time 1		Time 2	
	Monolingual (*n* = 37)	Bilingual (*n* = 41)	Monolingual (*n* = 27)	Bilingual (*n* = 25)
Age (months)	47.82 (6.94)	49.19 (7.32)	57.22 (6.68)	56.97 (5.25)
Gender	16 F, 21 M	19 F, 22 M	10 F, 15 M	9 F, 13 M
SES	29.64 (4.5)	28.02 (5.5)	-	-
Parental Education ^	5.24 (0.98)	5.0 (1.14)	-	-
Executive Function Composite	−0.27 (1.99)	0.26 (2.20)	0.48 (2.17)	−0.53 (1.76)
Happy/Sad task	10.30 (3.57)	11.56 (3.82)	12.26 (3.21)	12.96 (3.48)
Bear/Dragon task	8.65 (1.84)	8.83 (1.96)	9.70 (0.99)	9.44 (1.05)
Card Sort task	4.05 (1.79)	4.20 (1.57)	5.48 (0.98) *	4.20 (1.56) *
Receptive Vocab (Conceptual)	62.89 (12.29) **	47.17 (12.91) **	73.59 (14.19) **	57.44 (12.34) **
English	-	41.24 (16.40)	-	52.70 (13.63)
Spanish	-	34.05 (13.72)	-	43.30 (18.62)
Bilingual Total Vocabulary	-	73.44 (23.74)	-	92.92 (28.10)

Note: * *p* < 0.01; ** *p* < 0.001; ^ Primary caregiver level of education.

**Table 4:** Partial correlations controlling for age and autoregressive effects between Time 1 and Time 2 executive functioning (EF) and receptive vocabulary for the bilinguals.

	Executive Functioning 2	Receptive (Conceptual) Vocabulary 2	Receptive Total Vocabulary 2
Bilinguals			
Executive Functioning 1	0.22	0.09	0.13
Receptive Conceptual Vocabulary 1	0.14	0.45 ^	0.15
Receptive Total Vocabulary 1	0.09	0.44 ^	0.47 *
Monolinguals			
Executive Functioning 1	0.15	0.24	-
Receptive Vocabulary 1	0.58 **	0.67 **	-

Note: ^ *p* < 0.10; * *p* < 0.05; ** *p* < 0.01.

## Data Availability

The data presented in this study are available on request from the corresponding author. The data are not publicly available due to original informed consent provisions.
